# Oxidative Stress Biomarkers in Exhaled Breath of Workers Exposed to Crystalline Silica Dust by SPME-GC-MS 

**Published:** 2016-09-29

**Authors:** Mahdi Jalali, Mohammad Javad Zare Sakhvid, Abdulrahman Bahrami, Nima Berijani, Hussein Mahjub

**Affiliations:** ^a^ Department of Occupational Health, Social Determinants of Health Research Center, Birjand University of Medical Sciences, Birjand, Iran Email:; ^b^ Department of Occupational Health, School of Public Health, Yazd University of Medical Science, Yazd, Iran; ^c^ Excellence Center for Occupational Health, Research Centre for Health Sciences, School of Public Health, Hamadan University of Medical Science, Hamadan, Iran; ^d^ Department of Biostatistics, School of Public Health, Research Centre for Health Sciences, Hamadan University of Medical Sciences, Hamadan, Iran

**Keywords:** Breath tests, Occupational exposure, Biomarkers, Oxidative stress, Silicosis, Volatile organic compounds

## Abstract

**Background:** Silicosis is considered an oxidative stress related disease that can lead to the development of lung cancer. In this study, our purpose was to analysis of volatile organic compounds (VOCs) in the exhaled breath of workers exposed to silica containing dust and compare peak area of these compounds with silicosis patients and healthy volunteers (smokers and nonsmokers) groups.

**Methods:** In this cross sectional case-control study, the exhaled breath of 69 subjects including workers exposed to silica (n=20), silicosis patient (n=4), healthy non-smoker (n=20) and healthy smoker (n=25) were analyzed. We collected breath samples using 3-liter Tedlar bags. The VOCs were extracted with solid phase micro-extraction (SPME) and analyzed by gas chromatography-mass spectrometry (GC-MS). Personal exposure intensity was measured according to NIOSH 7601 method. Respiratory parameters were measured using spirometry.

**Results:** Seventy percent and 100% of the exposures to crystalline silica dust exceeded from 8 h TWA ACGIH TLVs in case and positive control groups, respectively. A significant negative correlation was found between dust exposure intensity and FEV_1_/FVC when exposure and positive control groups were studied in a group (r^2^ =-0.601, *P*<0.001). Totally, forty VOCs were found in all exhaled breath samples. Among the VOCs, the mean of peak area acetaldehyde, hexanal, nonanal, decane, pentad cane, 2-propanol and 3-hydroxy-2-butanone were higher in exhaled breath of the workers exposed to silica and silicosis patient compared to the healthy smoker and nonsmoker controls. In some cases the difference was significant (*P*<0.05).

**Conclusions:** The analysis of some VOCs in exhaled breath of subjects is appropriate biomarker to determine of exposure to silica.

## Introduction

Crystalline silica as a significant industrial material can cause silicosis in worker exposures^1^. Silicosis is considered an oxidative stress related disease that can lead to the development of lung cancer due to the genotoxic and fibrogenic effects of silica and its potential to produce oxidative stress. Silicosis develops increasingly and irreversibly over decades and there is no knowledge about cure of it^2^. The International Agency for Research on Cancer (IARC) and American Conference of Industrial Hygienists (ACGIH) classified crystalline silica (quartz and cristobalite) as a human carcinogen in 1997 (IARC, 1997) and as “suspected human carcinogen” A2 (ACGIH, 2000, 2004), respectively^3,4^.

Several methods have been developed for determining of crystalline silica in different types of products. The most important of these methods are: X-Ray diffraction method, infrared method and ultraviolet/visible Spectroscopy method^5^. Among all the analytical techniques, X-ray diffraction methods have the greatest potential for accurately identifying the polymorphs of crystalline silica. Infrared methods (IR) are the most promising for quantitative analysis of quartz in bulk materials^5^. In 1984 NIOSH issued analytical method 7601 for silica, crystalline that using the phosphoric acid digestion, followed by dissolving the residual silica, forming colored complexes and analyzing them by ultraviolet/visible spectroscopy^6^. This method can be used for respirable dust samples of less than 10 mm particle size and for determining quartz and cristobalite quantitatively in many industrial such as foundry that the digestion-resistant amorphous silica’s and silicates are not exist^5,7^.

When silica particles reach the alveoli; reacts with lung cells, leading to peroxidation of membrane lipids and damage to cell membranes. Silica dusts stimulate the generation of reactive oxygen species (ROS) either directly (on the particle surface) or indirectly (produced by the cell as a response to silica), which overwhelms antioxidant defenses of the lung and causes cell damage^8,9^. The main targets of free radicals are lipids, especially polyunsaturated fatty acids. Damage of membrane lipids substantially affects biological functions and/or the stability of cells. During the chain reactions, lipid peroxidation produces a variety of endogenous volatile compounds. Increase in concentrations of these compounds was correlated with development of oxidative stress in the body ^2,10^.

Currently, there are several clinical examinations for diagnosis of silicosis such as measurement of pulmonary function (Spirometry test), and grading of profusion according to the ILO classification of pneumoconiosis; however, no markers were found routinely to use for this disease progression yet^11^. Exposure biomarker is measured in the human body to assess exposure and defines as a chemical or its metabolite, or the product of an interaction between a chemical and some target molecule or cell. A biomarker should be ideal provided obtained easily with lowest uneasiness and minimum risk to the patient^12^.

One of the ways that recently considered for studying biomarkers of exposure and disease in toxicology, occupational medicine and assessment of oxidative stress, is exhaled breath analysis. Advantages of breath analysis in comparison with other biological samples such as blood and urine are its non-invasive matures, acceptable by patients or healthy volunteers, short time needed, high repeatability and easy to use^13^. Typical analysis techniques for exhaled breath are: 1) gas chromatography coupled with mass spectrometry (GCMS) 2) proton transfer reaction-mass spectrometry (PTR-MS) and 3) and selected-ion flow-tube mass spectrometry (SIFT-MS). Among these methods, GC-MS due to provide more details of exhaled breath is the most applicable^13^. Because of low concentration of volatile compounds in exhaled breath, quantification of these materials required a pre-concentration step. For this, solid phase micro extraction (SPME) method is more applicable, because of its simplicity; rapidity and elimination of chemical during the preparation stage^14-16^.

So far some researchers analyzed pollutants in workplaces and improving of inspection of occupational health^17,18^ and some studies investigated the non-volatile markers of oxidative stress (8-isoprostane, leukotriene's and malondialdehyde) in the exhaled breath condensate (EBC) of workers exposed to silica containing dust^19,20^.

To the best of our knowledge, there is no similar study for assessment of volatile markers associated with oxidative stress in the exhaled breath (gaseous Matrix) of workers exposed to silica containing dust using solid-phase micro extraction method. In this study, our propose was to analysis VOCs in the exhaled breath of workers exposed to dust containing silica and compare peak area of this compounds with positive control (silicosis patients) and healthy volunteers (smokers and nonsmokers) groups.

## Methods

### Design Study

This cross sectional case-control study was performed to evaluate exhaled breath of 69 individual male in Iran during March to October 2014.

### Demographics

This study was approved by Ethics Committee of Hamadan University of Medical Sciences, Hamadan, Iran. All the participants filled out the informed consent and signed it.

The sample size was calculated based on the concentration of exhaled breath hexanal (as one of the potential compounds associated with oxidative stress) in three groups of patients with lung cancer, healthy non-smokers and healthy smoker’s people, using following equation (α=0.05 and β=0.01) and according to the study of Poli and colleagues^21^, sample size estimated 20 people in each group.

Sample size (n) = (Z_1-α/2_ + Z_1-β_)^2^ + (σ_1_^2^+ σ_2_^2^) / (µ_1_+ µ_2_) ^2^

The subjects were asked to disclose their demographic and occupational characteristics, smoking habits and medical history in a questionnaire form. The exposed group consisted of 20 workers having five year working experience or more in 2 casting workshop. The positive control group consisted of 4 silicosis patients (two patients had retired and two others were transported to portion of without silica). A chest radiograph with an International Labor Office (ILO) classification of ≥1.0 in an individual with a history of silica dust exposure is definition of silicosis. {International Agency for Research on Cancer (IARC), 1997 #145}{International Labour Organization (ILO), 2011 #158}{ACGIH TLV, 2010 #55}{International Labour Organization (ILO), 2011 #158}{International Labour Organization (ILO), 2011 #158}The negative control group consisted of 20 healthy volunteer employed in office work, without any occupational exposure to dust, history of asthma, smoking and lung disease in the past 24 months. This data obtained from the medical files and self-declaration of the volunteers.

Six subjects of exposed group and three silicosis patients were smokers. Smoking habits could influence on detected compounds in exhaled breath. To deal with this bias, we selected smoker group too. The healthy smoker group consisted of 25 people employed in offices, smoking at least five cigarettes per day with history of smoking for at least one year and no occupational exposure to dust, history of asthma, acute and chronic lung diseases and cancers.

Smoking was measured as pack-years (number of cigarette packs smoked per day × number of years smoking).The subjects were not on any special diet or regime and did not consume any food, alcohol or cigarettes at least two hour before breath sampling.

### Spirometry Test and Inhalation Exposure Assessment

All spirometry measurements were performed using an auto calibrated flow-type spirometer (Spirolab III, Mir, Italy) according to the guidelines of American Thoracic Society/European Respiratory Society (ATS/ERS)^22^. Each test was repeated 3 times and the highest reading was taken for calculation. The following parameters recorded: forced expiratory volume in second (%FEV_1_) and forced vital capacity (%FVC). The FEV_1_/FVC ratio was calculated as percentage. Exposure of case group subjects to crystallinesilica was carried out according to the National Institute for Occupational Safety and Health (NIOSH), manual of analytical method 7601^6^. Personal respirable dust was sampled using a SKC pump (Model -224-PCXR3) with a flow rate of 1.7 L/min. A Rota meter was used to adjust the flow. The respirable dust samples were collected in 37-mm polyvinyl chloride (PVC) filter (pore size 5 µm) which placed in a 10 mm nylon cyclone. The cyclone was attached to the worker’s overalls as closely as possible near to the face in order to determine respirable dust in the breathing zone. The filters were conditioned in desiccator’s environmental chamber for 24 h at 25 °C and weighed before and after testing to determine total penetrating weights. For determining the crystalline silica in the samples, quartz standards were prepared and calibration curves were plotted. After preparation of the samples, then; crystalline silica was measured (in mg/m^3^) using of visible absorption Spectrophotometry in 420 nm wavelength. Exposure of silicosis patients (positive control group) to crystalline silica was obtained from medical files in the last year of exposure.

### Chemicals

Pentadecane, 2-heptanone, acetic acid, heptanoic acid, acetaldehyde, acetone, 2-propanol, acetonitrile, benzene, toluene, ethyl benzene, xylene, styrene, carbon disulfide, trichloromethane (>95%) were obtained from Merck (Darmstadt, Germ any). Hexanal, nonanal (95.0%) were obtained from Sigma–Aldrich (Milan, Italy). The SPME Carboxen/PDMS fiber and manual holders were supplied from Supelco (Bellefonte, PA, USA). Transparent Tedlar bags were obtained from SKC (Eighty Four, PA, USA).

### Breathe Gas Sampling

Alveolar breath gas was collected into 3-liter Tedlar bags which cleaned with purity nitrogen. Due to limitation in supply CO_2_ controlled device, a method was developed for the alveolar breath gas sampling. At this method, the subjects were requested to perform a slow vital capacity breath at a normal/constant flow without hyperventilating. After exiting the breath (exit of dead-space air) for five seconds, without doing a new respiration, exhaled breath remaining through a straw attached to bag transferred into bag for collecting alveolar breath gas. Multiple breaths (two or three exhaled breath) were performed for collecting 500 ml alveolar breath gas for each subject. After resting about for 10 min, the breath gas samples were obtained. In parallel, room air was obtained for background correction. All breathe gas samples were processed within 1-4 h after sampling. The last tobacco smoking was not processed shorter than 2 h before sampling.

### Extraction and Analysis

The VOCs in exhaled breath and ambient air were pre-concentrated by solid phase micro extraction (SPME) using 75 μm Carboxen/PDMS coated fibers (Supelco, Bellefonte, PA, USA). This method has recently been widely used in the extraction of VOCs in exhaled breath^23,24^. Before the first utilization, new fibers needed an initial preconditioning at a specified temperature and duration according to manufacturer’s instructions. Therefore, after assembling the fiber in the SPME device, the fiber was withdrawn and transferred into the injection port of the GC for 30 min in 250 ºC. In pre-concentration procedure, SPME needle was inserted into 3 liter Tedlar bag and fiber exposed with exhaled breath sample to VOCs extraction. Adsorption time was 20 min at 50 °C. Afterwards, the fiber was withdrawn and transferred into the injection port of the GC. Desorption time was 2 min while the temperature of the injection port was set at 290 °C.

The analysis was performed using Varian 3800 GC with a capillary column (RTX 624 with 25 m 0.25 mm 0.25 mm) equipped with a Saturn 2200 MS. The carrier gas was helium (99.999%) with flow rate of 1.5 ml/min. Split less mode was used. The injection port temperature was 290 ºC. The column temperature program was set at 35 ◦C initially and held at this temperature for 2 min, then increased to 140 ºC at 6 ºC/min and held for 5 min, finally increased to 200 ºC at 5 ºC/min and held for 3 min. The chromatographic run was completed in 35 min. The MS analyses were carried out in a full scan (scan range 10–600 amu) for all samples. Ionization energy of 70 eV was applied. Some of the compounds were identified by its mass spectrum and for the some others identification was confirmed by comparing the retention times and mass spectra with those pure standard of substances. The chromatographic data acquisition was performed with NIST 05 library software.

### Statistical analysis

Data were analyzed using SPSS (Chicago, Illinois, USA). The distribution of parameters was examined with Shapiro Wilk test. The mean values of spirometric parameters were compared by Kruskal–Wallis H test between exposed, positive and negative control groups. One way ANOVA on the rank-transformed data and Tukey post hoc were employed to detect significant differences between VOCs detected in the groups. Independent two-sample t-test was conducted for compare mean exposure intensity with crystalline silica dust in exposed and positive control groups. In all tests, the level of significance was set at *P*< 0.05.

## Results

### Subject’s characterization

Occupational and demographic characteristics of case group, silicosis patients, healthy volunteers and healthy smokers are presented in Table 1. Statistically significant differences were observed in the mean age of the groups (*P*=0.046). Groups were matched together in term of height, weight and BMI. Besides, the work experience was equaled in the exposed and negative control groups and different in the positive control with other groups. Six exposed groups and three silicosis patients had a history of cigarette smoking. The mean (standard deviation) cigarette smoking in the exposed, positive control and healthy smokers groups were 3.51 (2.71), 5.16 (0.76) and 2.98 (3.54) packs-years respectively.

**Table 1:** Demographic characteristics of study groups

**Groups**	**Mean**	**SD**	**Min**	**Max**	***P *** **value**
Age (yr)					0.046
Exposure	42.7	8.5	28.0	56.0	
Negative control	41.4	6.9	28.0	55.0	
Positive control	51.0	4.9	45.0	57.0	
Smokers	40.9	8.4	27.0	58.0	
Height (cm)					0.404
Exposure	177.5	4.2	168.0	182.0	
Negative control	176.2	5.2	170.0	188.0	
Positive control	174.5	4.0	170.0	177.0	
Smokers	175.2	4.6	169.0	185.0	
Weight (kg)					0.067
Exposure	79.1	8.6	66.0	100.0	
Negative control	81.3	8.1	65.0	95.0	
Positive control	78.5	5.3	71.0	82.0	
Smokers	80.2	7.9	66.0	94.0	
Body mass index (kg/m^2^)					
Exposure	25.1	2.5	20.3	30.8	0.054
Negative control	27.1	2.5	22.4	30.0	
Positive control	25.4	1.4	22.7	25.0	
Smokers	26.1	2.5	21.3	30.0	
Work experience (yr)					0.012
Exposure	18.4	7.5	6.0	29.0	
Negative control	17.9	8.7	5.0	25.0	
Positive control	26.0	3.9	23.0	30.0	

### Silica exposure and spirometric results

Table 2, summarizes the performed spirometry tests (during a period of 12 months, from April to July 2014) and Inhalation exposure intensity (mg/m^3^) in the studied groups. Significant statistically differences were observed at the mean FEV_1_/FVC%, FEV_1_ and FVC% among the groups (*P*-value<0.05).

**Table 2:** Pulmonary function test data and Inhalation exposure intensity (mg/m^3^) in study groups

**Groups**	**Mean**	**SD**	**Min**	**Max**	***P*** ** value**
FEV_1_ (%)					
Exposure	103.5	16.5	81.0	126.0	0.002
Negative control	90.8	10.1	74.5	110.0	
Positive control	74.5	1.9	72.0	76.0	
FVC (%)					
Exposure	120.2	25.6	86.0	164.0	0.001
Negative control	91.3	7.7	77.0	110.0	
Positive control	113.5	5.2	109.0	121.0	
FEV_1_/FVC (%)					
Exposure	71.6	5.7	64.0	84.0	0.001
Negative control	83.4	2.7	78.0	88.0	
Positive control	60.7	2.7	57.9	64.0	
Respirable dust					
Exposure	5.2	1.8	1.8	8.2	0.026
Positive control	7.5	1.1	5.9	8.5	
Crystalline silica					
Exposure	0.1	0.1	0.1	0.3	0.006
Positive control	0.2	0.1	0.2	0.3	

The mean values of crystalline silica dust exposure were 0.115 mg/m^3^ (SD=0.080) and 0.245 mg/m^3^ (SD=0.059) for case and positive control groups, respectively. Seventy percent and 100% of the exposures to crystalline silica dust in the personal samples exceeded from 8 h TWA ACGIH TLVs (0.025 mg/m^3^) in case and positive control groups, respectively. There was no significant correlation between pulmonary function tests and silica exposure. Anindirect significant correlation was found between dust exposure intensity and FEV_1_/FVC when exposure and positive control groups were studied in a group (r^2^=-0.601, *P*<0.001).

### Exhaled breath measurements

**Figure 1:**
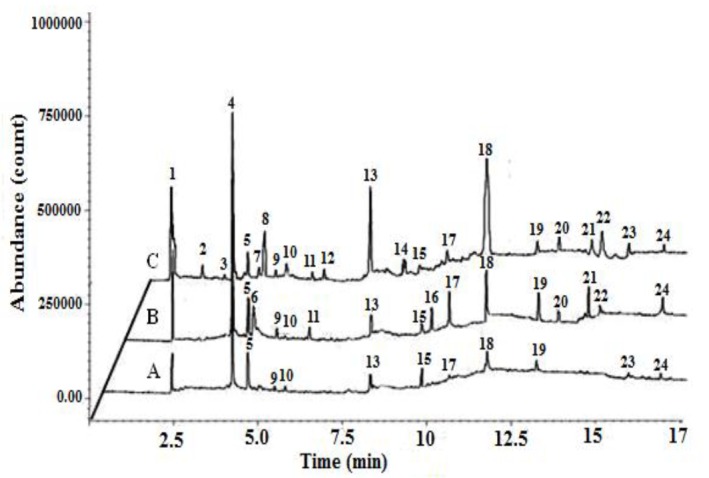
The GC-MS chromatogram of exhaled breath from a negative control (A), worker exposed to silica containing dust (B) and healthy smoker (C)

The frequency of identified VOCs in exhaled breath of case, positive control, negative control and healthy smoker groups are presented in Table 3. Totally, forty VOCs were found in allbreath samples (at least once). Terpinolen (contained in foodstuff), phenol and N, N-dimethyl acetamide (released from Tedlar bags) and carbon disulfide (released by GC-MS septa frequently) were excluded. Twenty compounds were identified by spectral library match. Isoprene, acetone, benzene and toluene were found in all samples. Alcohols, alkanes and methylated alkanes, unsaturated aldehydes, ketones, unsaturated hydrocarbons, nitrogen-containing compounds, non-cyclic alkenes, benzene derivatives, volatile acids and furans were among the identified VOCs. Figure 1 shows an example of GC / MS chromatogram of exhaled breath of healthy nonsmokers, healthy smokers and workers who exposed to silica containing dusts. Differences in the type, number and peak area of detected VOCs in exhaled breath is clear.

**Table 3:** The frequency of identified VOCs in exhaled breath of study groups (percentage of observations in each group)

**Variables**	**Exposure**	**Negative control**	**Positive control**	**Smoker**	**Total**
**Acetaldehyde** ^a^	15 (75)	13 (65)	4 (100)	19 (76)	51 (74)
2-methyle propane	-	9 (45)	2 (50)	13 (52)	24 (35)
Ethylamine	1 (5)	2 (10)	2 (50)	9 (36)	14 (20)
2-methyle 1-proanol	-	3 (15)	-	-	3 (4)
2-pentene	-	3 (15)	2 (50)	13 (52)	18 (26)
Isoprene	20 (100)	20 (100)	4 (100)	25 (100)	69 (100)
**Acetone** ^a^	20 (100)	20 (100)	4 (100)	25 (100)	69 (100)
**2-propanol** ^a^	12 (60)	3 (15)	3 (75)	3 (12)	21 (30)
1-3 cyclopentadiene	-	-	2 (50)	10 (40)	12 (17)
1-3 cyclohexadiene	-	2 (10)	-	7 (28)	9 (13)
Decane	16 (80)	6 (30)	3 (75)	8 (32)	33 (48)
**Acetonitrile** ^a^	6 (30)	3 (15)	3 (75)	18 (72)	30 (44)
3-methyle pentane	-	3 (15)	1 (25)	7 (28)	11 (16)
Butene	15 (75)	15 (75)	3 (75)	15 (60)	48 (70)
1-3 butadiene	12 (60)	-	3 (75)	16 (64)	31 (45)
2-4 hexadiene	4 (20)	-	-	17 (68)	21 (30)
**3-chloro methane** ^a^	-	2 (10)	-	-	2 (3)
Octane	-	5 (25)	2 (50)	11 (44)	18 (26)
**Benzene** ^a^	20 (100)	20 (100)	4 (100)	25 (100)	69 (100)
**Acetic acid** ^a^	8 (40)	9 (45)	3 (75)	8 (32)	28 (40)
2-5 dimethyl furan	4 (20)	-	3 (75)	18 (72)	25 (36)
Propanthiol	20 (100)	13 (65)	3 (75)	13 (52)	49 (71)
3-hydroxy 2-butanone	19 (95)	11 (55)	1 (25)	8 (32)	39 (56)
**Heptanoicacid** ^a^	19 (95)	12 (60)	2 (50)	20 (80)	53 (77)
Nitro propane	-	-	1 (25)	-	1 (2)
**Toluene** ^a^	20 (100)	20 (100)	4 (100)	25 (100)	69 (100)
**Hexanal** ^a^	20 (100)	11 (55)	4 (100)	16 (64)	51 (74)
**Pentadecane** ^a^	20 (100)	12 (60)	2 (50)	13 (52)	47 (68)
Butanoic acid	14 (70)	-	3 (75)	17 (68)	34 (50)
**Ethyl benzene** ^a^	5 (25)	-	3 (75)	6 (24)	14 (20)
**Xylene** ^a^	5 (25)	-	3 (75)	11 (44)	19 (27)
2-heptanone	-	1 (5)	-	2 (8)	3 (4)
**Styrene** ^a^	2 (10)	3 (15)	3 (75)	6 (24)	14 (20)
Furan methanol	4 (20)	-	-	1 (4)	5 (7)
Dimethyl benzene	1 (5)	8 (40)	3 (75)	12 (48)	24 (35)
**Nonanal** ^a^	12 (60)	3 (15)	4 (100)	10 (40)	29 (42)

^a^ Identify by comparing the pure standard retention times and mass spectrumin NIST library

Statistically significant differences among the mean peak area of identified VOCs in exhaled breath study groups are presented in Table 4. Statistically significant differences were observed in the mean peak area of acetaldehyde, 2-propanol, decane, 1,3 butadiene, propanthiol, 3-hydroxy-2-butanone, hexanal, pentadecane, butanoic acid and nonanal in the exhaled breath of the exposure and negative control groups (*P*<0.05). Therefore, the mean peak area of these VOCs in the exhaled breath of exposure group was higher than negative control group.

**Table 4:** Statistically significant differences among the mean peak area of identified VOCs in exhaled breath study groups

**Pair groups **	**C-N**	**C-P**	**C-S**	**N-P**	**N-S**	**P-S**
Acetaldehyde	0.026	0.050	0.048	0.003	0.801	0.015
2-methyle propane	0.014	0.232	0.001	0.999	0.912	0.995
Ethylamine	0.987	0.068	0.038	0.105	0.087	0.741
2-methyle 1-proanol	0.088	1.000	1.000	0.515	0.066	1.000
2-pentene	0.582	0.056	0.001	0.259	0.030	0.991
Isoprene	0.046	0.984	0.032	0.042	0.987	0.034
Acetone	0.256	0.792	0.042	0.247	0.771	0.078
2-propanol	0.001	0.237	0.046	0.003	0.888	0.001
1-3 cyclopentadiene	1.000	0.021	0.001	0.021	0.001	0.855
1-3 cyclohexadiene	0.802	1.000	0.023	0.953	0.204	0.366
Decane	0.004	0.713	0.011	0.765	0.958	0.983
Acetonitrile	0.712	0.173	0.001	0.050	0.001	0.970
3-methyle pentane	0.548	0.610	0.049	0.967	0.997	0.998
Butene	1.000	0.852	0.991	0.848	0.793	0.761
1-3 butadiene	0.001	0.999	0.987	0.035	0.001	0.985
2-4 hexadiene	0.437	0.815	0.001	1.000	0.001	0.004
3-chloro methane	0.238	1.000	1.000	0.693	0.198	1.000
Octane	0.269	0.111	0.004	0.607	0.368	0.986
Benzene	0.662	0.236	0.031	0.049	0.001	0.983
Acetic acid	0.788	9.468	0.968	0.789	0.479	0.308
2-5 dimethyl furan	0.452	0.048	0.001	0.005	0.001	1.000
Propanthiol	0.001	0.184	0.001	0.944	0.439	0.499
3-hydroxy 2-butanone	0.006	0.003	0.001	0.359	0.037	0.999
Heptanoic acid	0.128	0.125	0.006	0.779	0.720	0.981
Nitro propane	1.000	0.001	1.000	0.001	1.000	0.001
Toluene	0.996	0.817	0.005	0.879	0.010	0.727
Hexanal	0.001	0.282	0.001	0.001	0.786	0.001
Pentadecane	0.001	0.015	0.001	0.993	0.515	0.720
Butanoic acid	0.001	1.000	0.999	0.027	0.001	0.999
Ethyl benzene	0.132	0.036	0.986	0.001	0.204	0.014
Xylene	0.351	0.009	0.204	0.001	0.002	0.012
2-heptanone	0.880	1.000	0.563	0.937	0.953	0.886
Styrene	0.976	0.010	0.681	0.019	0.905	0.044
Furan methanol	0.077	0.493	0.187	1.000	0.945	0.990
Dimethyl benzene	0.015	0.002	0.007	0.050	0.760	0.021
Nonanal	0.003	0.038	0.047	0.001	0.512	0.001

C: Exposure group N: Negative control P: Positive control S: Smoker group

The mean peak area of acetaldehyde, isoprene, 2-propanol, acetonitrile, benzene, hexanal, styrene, dimethyl benzene and nonanal were increased in the exhaled breath of positive control compared to those of negative control group (*P*<0.05).

Statistically significant differences were observed in the mean peak area of acetaldehyde, 2, 5 dimethyl furan, ethyl benzene, xylene, styrene, dimethyl benzene and nonanal in the exhaled breath of case and positive control groups (*P*<0.05). Therefore, the mean peak areas of these compounds in the exhaled breath of positive control were higher compared to those of case group.

(1) acetaldehyde, (2) 2-methyl propane, (3) 2-pentene, (4) isoprene, (5) acetone, (6 )2-propanol, (7) 1-3 cyclohexadiene, (8) acetonitrile, (9) decane, (10) butene, (11) 1-3 butadiene (12) 2-4 hexadiene, (13) benzene, (14) 2-5 dimethyl furan, (15) propanthiol, (16) 3- hydroxyl 2-butanone, (17) heptanoic acid, (18) toluene, (19) hexanal, (20) butanoic acid, (21) ethyl benzene, (22) xylene, (23) styrene, (24) nonanal.

The mean peak area of ethylamine, acetonitrile, 2, 4 hexadiene, benzene, 2, 5 dimethyl furan, toluene, hexanal and dimethyl benzene were increased in the exhaled breath of healthy smokers compared to those of case group and statistically difference were significant (*P*<0.05).2 methyl propane, 2 pentene, 1, 3 cyclopentadiene, 1, 3 cyclohexadiene, decane, 3 methyl pentane and octane none detected in the exhaled breath of case group, but were detected in the healthy smokers. The mean peak area of isoprene was lower in the exhaled breath case and positive control groups than the smoker and nonsmoker volunteer groups and statistically difference were significant (*P*<0.05).

## Discussion

The obtained results of current study demonstrated difference feature between the exhaled breath of workers exposed to silica containing dust and silicosis patient with those of the smoker and healthy controls.

Surveying demographic and job characteristics in the studied groups showed that the mean age and work experience were similar with the exception of the positive control group. The mean weight, height and body mass index were similar in all of the surveyed groups. Therefore, the effect of variables such as weight, height and body mass index was eliminated in all of the groups. The effect of age among the negative control, exposed and smoker groups and effect of work experience was eliminated from the negative control and exposed groups as well. The only difference was between age and work experience groups with positive control group.

The results of this study showed difference in dust exposure intensity and changes in respiratory parameters among the studied groups. Exposure to silica containing dust in all silicosis patients and more of exposed workers group was set higher than the occupational exposure limits. These results are consistent with previous studies that proved the excessive exposure to silica containing dust in casting workshops workers^7,25^. Moreover, changes in respiratory parameters in exposed and silicosis patients groups were founded. When the groups were studied separately, there was no significant correlation between respiratory parameters and the dust exposure intensity. But when the exposure and silicosis patient groups were studied as a group, a significant correlation was observed between FEV_1_/FVC and intensity of exposure to silica containing dust indirectly. Bahrami and Colleagues^26^ and Wang and Colleagues^27^ in separate studies, showed that exposure to silica containing dust reduce respiratory parameters such us FVC, FEV_1_ and FEV_1_/FVC, and an indirect correlation between FEV_1_/FVC and exposure intensity to silica containing dust is in line with the results of these studies. After studying the exposed and positive control groups separately, lack of correlation between spirometry parameters and intensity of exposure to silica containing dust was found that might be due to the small sample size. It can be considered as one of the limitations of the present study.

### VOCs Related to Smoking

Obtained results demonstrate that the mean peak area of some compounds such as acetonitrile, 3-methylpentane, 2, 5-dimethyl furan, toluene, benzene, xylene, styrene, 1, 3-cyclopentadiene and dimethyl benzene were higher in the exhaled breath of healthy smokers and positive controls compared to those of other groups. Consequently, because of the fact that the three silicosis patients (75%) consume cigarettes with 35-38 pack in years, thus these VOCs may be correlated with smoking. The smoking-related origin of acetonitrile, 2, 5-dimethyl furan, toluene, benzene, xylene and styrene have been supported in previous studies^28^. In addition, the presence of compounds including benzene, xylene and styrene in the exhaled breath can be associated with environmental contaminants. Presence of dimethyl benzene as a benzenoid compounds in the exhaled breath seems to be resulted from air pollution^29^. Furthermore, presence of 1, 3-cyclohexadiene, 1, 3-cyclopentadiene, 2-methyl-1-butene, 2, 4-hexadiene and 2-pantene in the exhaled breath are associated with smoking habits^29,30^.

### VOCs Related to Lipid Peroxidation

Silica particles are leading cause of activation of reactive oxygen species (ROS) after reaching to lung parenchyma and probable phagocytizing by lung macrophages^8^. These produced ROS as free radicals may influence and damage cell membranes and genetic structure of different organs particularly the related lung cells^9^. The consequent effect of these free radicals on polyunsaturated fatty acids and lipids of cell membranes is induction of lipid peroxidation^10^. Several types of volatile compounds such as aldehydes, alkanes and methylated alkanes are yielded by lipid peroxidation during the chain reactions, which are either metabolized or excreted in the breath^30^.

Our results showed that mean of peak area of compounds including acetaldehyde, hexanal, and nonanal were higher in exhaled breath of exposure and positive control groups than negative control and healthy smoker groups. The elevated levels of aldehydes are considered as the biomarker for enhanced oxidative stress. Peroxidation of ω3 and ω6 fatty acids (PUFAs) as the basic components of cell membrane phospholipids leads to form saturated aldehydes such as hexanal and nonanal particularly^21^. It may be thought that the higher peak area of hexanal and nonanal in exhaled breath of exposure and positive control groups compared to two other studied groups is associated with membrane lung injury caused by silica particles and creating oxidative stress, as described previously. The aforementioned finding is consistent with results of Fuchs et al., who detected significantly higher concentrations of hexanal and nonanal in exhaled breath of lung cancer patients compared to those of smokers and healthy controls^31^. The concentration of acetaldehyde is always much lower than ethanol, thus the origin of acetaldehyde found in normal human breath possibly results from the oxidation of endogenously produced ethanol. Surprisingly, in current study ethanol was not found in any of the samples. Presence of acetaldehyde in breath is associated with air pollution, tobacco smoke and alcohol metabolism^21^. Therefore, the exact role of this compound as a specific marker of oxidative stress is unclear, until now.

Alkanes and methylated alkanes are known as lipid peroxidation marker^32^. In current study, mean of peak area of decane was higher in exhaled breath of exposure group compared with that of all control groups. In addition, its mean of peak area was higher in exhaled breath of positive control group compared to that of healthy smoker and nonsmoker groups. This finding is in agreement with another study on individuals exposed to asbestos^33^. Accordingly, the decane level was higher in exhaled breath of exposed group compared to that of positive (patients with malignant pleural mesothelioma) and negative (healthy subjects) control groups. Pentad cane, the other alkane, also was detected in the 100% of exhaled breath of exposure group and its mean of peak area was higher in exhaled breath exposure group compared to all control groups. There was significant difference between exposure and negative control groups and between exposure and smoker groups. Pentad cane increased in the breath of women with breast cancer^34^. In the current study, obtained results about increase in the mean of peak area decane and pentad cane in the exhaled breath of exposure and positive control groups can be associated with oxidative stress induced from exposure to crystalline silica and lipid peroxidation in the lung cell membranes, as previously described.

Bajtarevic et al and Song et al. have reported 3-hydroxy-2-butanone, a ketone, as lung cancer markers in exhaled breath^24,30^. In current study, the mean of peak area of 3-hydroxy-2-butanone was higher in the exhaled breath of exposure group compared to all control groups. However, its mean of peak area in the exhaled breath of negative control group was higher compared to positive control and smoker groups. At present, there is no exact knowledge about biochemical pathway for the production of endogenous 3-hydroxy-2-butanone in the human body associated with oxidative stress. The concentration of 3-hydroxy-2-butanone in the exhaled breath of cancer patients was higher than that of controls associated with oxidative products of butane^24^. Increase in 3-3-hydroxy-2-butanone level is directly associated with increase in oxidative activity. In aforementioned study, the mean of peak area of 3-hydroxy-2-butanone was lower in exhaled breath of positive control compared to that of negative control and smoker groups; which is in contrast to result of current study.

In current study, the mean of peak area of 2-propanol was higher in exhaled breath of exposure and positive control groups compared to that of negative control group. Therefore, the difference was significant statistically. Concentrations of 2-propanol in the exhaled breath of breast cancer and lung cancer patients was significantly higher than that of healthy controls^35,36^. There is association between presence of 2-propanol in exhaled breath and the ambient air particularly in a clinical environment^29,30^. Until now, it was not suggested any biochemical pathways for the production of endogenous 2-propanol which is associated with oxidative stress.

Isoprene is one of the most common VOCs found in exhaled breath in the highest concentration^37^. Considering isoprene as a marker of oxidative stress remains controversial. Isoprene may reflect the oxidative stress indirectly^38^. In the current study, the mean of peak area of isoprene in the exhaled breath of exposure and positive control groups was lower than that of smoker and nonsmoker volunteer groups. This fact could be associated with lower exhalation force in exposure and positive control groups compared to that of other control groups^39^. Based on the study by Bajtarevic et al. on patients with lung cancer, low isoprene has observed in patients compared to that of healthy individuals^30^.

There were several limitations in performing current study. We had limitation of providing CO_2_ controlled device; thereby we applied simple method for collecting alveolar air that was based on control of time of dead air exist from respiratory duct. This applied method probably had low sensitivity and could cause errors in current study. Somehow, in order to decrease probable errors, accompany by collecting the exhaled breath, the ambient environmental air was collected too; and finally amount of discovered compounds in ambient environmental was subtracted from similar discovered compound in exhaled breath. In the current study, we used the peak area for quantification, which is insufficient to quantify correctly. It was better that analysis be performed by comparing the peak area of the volatile compounds with the peak area of the calibration series run together. However, because of time and financial constraints we aren’t able to quantification the compounds were detected in exhaled breath. Enrolling low sample size in groups and lack of investigate influence of some factors such as the type of task and activity sites of workers in air samples collection can be also considered as another limitation of current study. Considering some smoker individuals in positive control and exposed groups, we enrolled a smoker group to eliminate error caused by consuming cigarette; in other words, smoking habits could influence on detected compounds in exhaled breath; to deal with this bias, we selected smoker group. In addition, to confirm differences in possible VOCs related to oxidative stress in exhaled breath of individuals exposed to silica containing dust and nonsmoker healthy individuals, we also enrolled several patients with silicosis (whose high level of oxidative stress have been observed in previous several studies) as positive control group. The current study is the first investigation about analysis of VOCs in the exhaled breath of individuals exposed to silica containing dust for discovering possible compounds related to oxidative stress. Consequently, with regard to mentioned limitations, these initial results are usable for current study and should be explained with caution.

## Conclusions

The results of current study confirmed difference of VOCs present in exhaled breath of individuals exposed to silica containing dust and individuals with silicosis with those of smoker and nonsmoker healthy individuals. It seems that the eight compounds including acetaldehyde, hexanal, nonanal, decane, pentad cane, 3-hydroxy-2-butanon, 2-propanol and isoprene in the exhaled breath of individuals exposed to silica containing dust and patients with silicosis could possibly be taken as useful breath biomarkers for exposure to silica containing dust. However, additional studies including simultaneous assessment of blood, urine, and exhaled breath markers are needed to confirm this topic.

## Acknowledgments

This research was part of MSC. Thesis at Hamadan University of Medical Sciences and authors thank for financial support (Grant no. 4913) for this research.

This study was approved by Vice-Chancellor of Education and funded by the Vice-Chancellor of Research and Technology, Hamadan University of Medical Sciences (No. 4913).

## Conflict of interest statement

The authors declare that they have no conflicts of interest.

## Highlights

- Exhaled breath analysis is a non-invasive and simple method for assessment of endogenous VOCs in occupational studies
- There are differences in the exhaled breath of workers exposed to silica dust with the healthy controls
- Study of some VOCs in exhaled breath of subjects is appropriate biomarker to determine of exposure to silica
